# An Insight to the Composition of Pre-Hispanic Mayan Funerary Pigments by ^1^H-NMR Analysis

**DOI:** 10.3390/molecules26102972

**Published:** 2021-05-17

**Authors:** Kadwin J. Pérez-López, Vera Tiesler, Patricia Quintana, Emanuel Hernández-Nuñez, Gloria I. Hernández-Bolio

**Affiliations:** 1 Facultad de Ciencias Antropológicas, Universidad Autónoma de Yucatán, Mérida 97305, Yucatán, Mexico; kadwinp@gmail.com (K.J.P.-L.); vtiesler@correo.uady.mx (V.T.); 2 Centro de Investigación y de Estudios Avanzados, Departamento de Física Aplicada del Instituto Politécnico Nacional, Unidad Mérida, Mérida 97310, Yucatán, Mexico; pquint@cinvestav.mx; 3 CONACYT, Centro de Investigación y de Estudios Avanzados del Instituto Politécnico Nacional, Departamento de Recursos del Mar, Unidad Mérida, Mérida 97310, Yucatán, Mexico

**Keywords:** abietane diterpenoids, beeswax, bone remains, resins

## Abstract

The funerary rites of particular members of the pre-Hispanic Mayan society included the pigmentation of the corpse with a red color. In order to understand this ritual, it is first necessary to identify the constituents of the pigment mixture and then, based on its properties, analyze the possible form and moment of application. In the present approach, ^1^H-NMR analysis was carried to detect organic components in the funerary pigments of Xcambó, a small Maya settlement in the Yucatan Peninsula. The comparison of the spectra belonging to the pigment found in the bone remains of seven individuals, and those from natural materials, led to the identification of beeswax and an abietane resin as constituents of the pigment, thus conferring it agglutinant and aromatic properties, respectively. The ^1^H-NMR analysis also allowed to rule out the presence of copal, a resin found in the pigment cover from paramount chiefs from the Mayan society. Additionally, a protocol for the extraction of the organic fraction from the bone segment without visible signs of analysis was developed, thus broadening the techniques available to investigate these valuable samples.

## 1. Introduction

It was mainly during the first millennium of our era that a range of funeral treatments were deployed in Maya lands. In a particular ceremony, the ancient settlers wrapped their deceased in color by adding red pigments in elaborate rituals that would ensure the following life. The red color had a profound symbolic significance amongst the pre-Hispanic Maya. Red coloring materials were present as part of their daily and ritual life, while mineral pigments and organic colorants were used to cover the skins of both living and dead individuals [1]. Archaeological evidence has revealed the use of red color on funerary rituals of distinguished members of the Maya society as the Red Queen of Palenque and the *Ahaw* Garra de Jaguar of Calakmul [2]; however, red pigments are also reflected in a more discreet way in simple burials of small communities. This is the case of the site of Xcambó (Yucatán) (Figure S1, in the Supplementary Material), a saline and merchant port of the past that stands out for the large number of men, women, and children that would have been pigmented and, in general, for the good state of conservation of its extensive bone collection (with more than five hundred individuals) [3]. This site, originally established as a salt production center, was continuously populated from the Early (AD 250–550) to the Late Classic (AD 550–700) period [4]. A fact that is remarkable from Xcambó is the number of pigmented individuals and their good state of preservation: the skeletal collection with 13.8% of the bones showing red pigmentation, constitutes one of the largest and better conserved in the Mayan area [1].

These individuals were pigmented in funerary events during the Late Classic, deposited in simple burials or constructions, distributed throughout the site, although mainly in the central plaza area. They also show complete body pigmentation and, on a few occasions, only the skull or head, a particularly symbolic area [5,6].

It has been suggested that the practice consisted of sprinkling pigments, smearing paints, or using painted wrappings on the body or skeletonized remains of the deceased, but in the absence of historical sources describing such events, little is known about the steps involved in the ritual. For this reason, the colorations preserved on the skeletons offer valuable paths for the study of the materials involved and the moments of their application.

The current knowledge about the composition of the red pigments from the bone remains found in Xcambó and other archaeological sites (Figure S2, in the Supplementary Material), is based on X-ray diffraction and electronic microscopy analyses of pigment samples, which have revealed the presence of mainly inorganic minerals, e.g., cinnabar (HgS), found in the volcanic lands and used only in elite rituals, and hematite (Fe_2_O_3_), a more accessible material commonly used by the general Maya population [7]. Recently, it has been reported that these minerals could be a part of a complex mixture which include gums, agglutinants, and aromatic components such as the copal resin [8]. Additionally, analyses of pigment samples using pyrolysis coupled with gas-chromatography/mass spectrometry (Pyr-GC-MS), has allowed the detection of a number of natural components, which include aromatic excipients, such as acacia gum, and copal and pine resins. However, the main disadvantages of Pyr-GC/MS are that is not possible to recover the sample and that only the volatile fraction of the sample is detected [9]. Nuclear magnetic resonance (NMR) represents a viable alternative to identify the components in pigments found in archaeological samples; in addition to being a non-destructive technique, it can, at the same time, aid in the identification of metabolites from a single profile [10]. To date, liquid state NMR has been successfully applied in the analysis of the organic components in bituminous materials, as well as wood, bone, leather, amber, and resins, among other archaeological samples [11,12], leading to the identification of chemical compounds that can provide information about the materials used by ancient people in everyday life, for tools and artifact construction, food preparation, diet, etc. [13]. NMR has also been applied to distinguish conifer exudates from those belonging to other resinous plant families based on the molecular structure of their components, allowing an straightforward classification using bulk resins [14]. In addition, particular ^1^H-NMR signals can guide to the identification of characteristic metabolites [15]. The aim of the present study was to evaluate the use of ^1^H-NMR as an analytical tool for the detection and characterization of the organic components present in the funerary pigment of bones found in Xcambó, Yucatan.

## 2. Results and Discussion

The first step in this study included the analysis of samples from different archaeological contexts of the site of Xcambó in order to detect the presence of organic components in the pigment mixture. The extraction of organics from the pigmented bones was evaluated using two methods, extraction of the bone with CH_2_Cl_2_ and direct scraping (Table 1).

**Figure 1:**
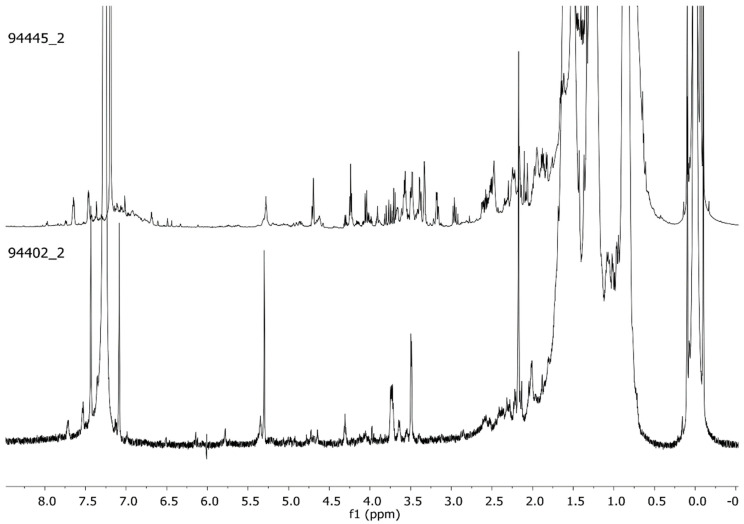
Representative ^1^H-NMR profiles (CDCl_3_, 600 MHz) of the red pigment’s organic fraction extracted from scraped bone (94402_2) and by sonication of the bone segment (94445_2).

Of the two, sonication of the bone segment with CH_2_Cl_2_, followed by concentration and resuspension in CDCl_3_, produced a better yield of extract as seen when comparing to additional samples of the same site (Figure S3, in the Supplementary Material). The sonication did not affect the bone surface or the pigment varnish (Figure 1). This is a plus that could allow the access to larger bone pieces to be explored without damage.

**Figure 2:**
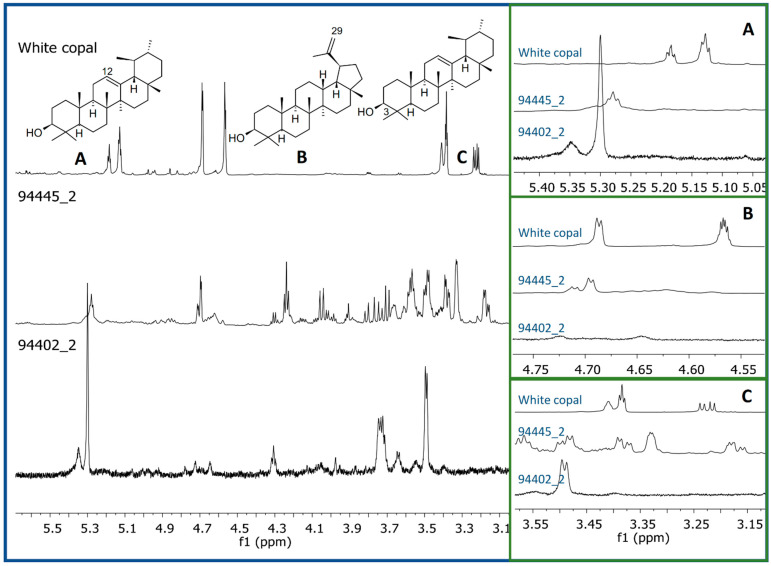
Comparison of the ^1^H-NMR profiles (CDCl_3_, 600 MHz) from two representative pigment samples (scraping and sonication) with those of white copal showing the characteristic signals: (**A**) H-12 from *α* and *β*-amyrin, (**B**) H-29 from lupanes, and (**C**) H-3 from *α* and *β*-amyrins.

The ^1^H-NMR profiles of the pigments found in the bone remains of seven individuals (male and female) from Xcambó site, and extracted by the two methods, showed several proton resonances corresponding to a variety of organic metabolites, which were present in all the samples. The most abundant signals were found in the aliphatic (δ0.50–2.00) and carbinol-proton regions (δ3.00–4.50) (Figure S3, in the Supplementary Material). Considering that, in the cases of the Red Queen of Palenque and the dignitary buried in the Structure III-9 at Calakmul, excipients with aromas of copal and pine were found in the funerary pigment [8], the ^1^H-NMR profiles of the funerary pigments analyzed in this study were compared with those of copal resin and commercial standards of its characteristic triterpenes [16]. None of the signals from the copal resin or its lupane and amyrin triterpenes, marker compounds of the *Bursera* species, were observed in the ^1^H-NMR profiles in the funerary pigments (Figure 2), ruling out the presence of copal in the different samples. It is possible that, similarly to the mineral composition, some differences there exist between Maya elites and popular social sectors, i.e., while dignitaries’ pigment include high value resins as copal, the funerary pigment of people from minor communities is constituted with more accessible materials; however, more studies in this type of individuals are necessary to confirm such hypothesis.

**Figure 3:**
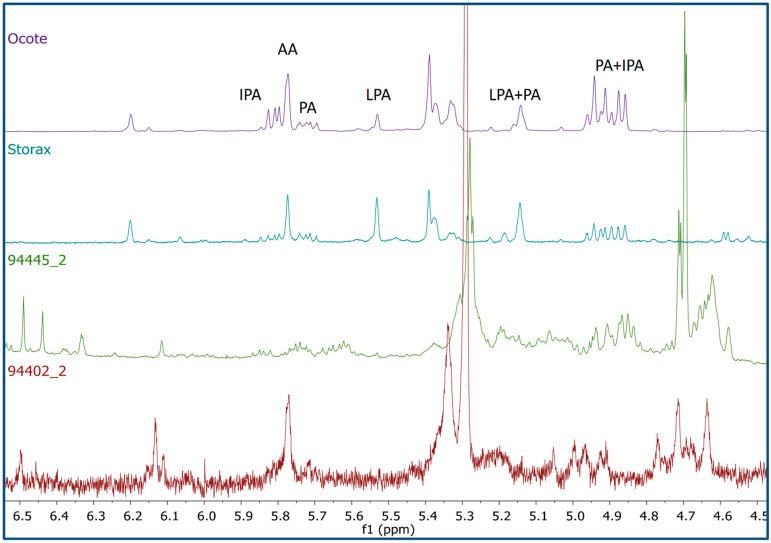
Comparison of the ^1^H-NMR profiles (CDCl_3_, 600 MHz) from two representative pigment samples (scraping and sonication) with those of ocote and storax resin showing some characteristic resonances from abietane diterpenes: levopimaric (LPA) (H-8, δ5.49; H-6, δ5.11), abietic (AA) (H-8, δ5.78), pimaric (PA) (H-13, δ5.72; H-8, δ5.15; H-14, δ4.95, and 4.91), and isopimaric (IPA) acids (H-14, δ5.81; H-15, δ4.93, and δ4.87).

A comparison of the ^1^H-NMR profile of the funerary pigments with various natural materials allowed the detection of a number of proton signals that were present in both the funerary pigments profiles and those from the resins of “ocote” (*Pinus* spp.), storax (*Styrax* spp.), and beeswax. Several of the signals originating from components in the resins appeared in the region of δ4.60–6.20 showing a chemical shift and coupling pattern which suggested the presence of levopimaric (LPA), abietic (AA), pimaric (PA), and isopimaric (IPA) acids [17] (Figure 3). These abietane diterpenes are major components of the resins of conifers and have been isolated mainly from Pinaceae species [18]. This finding suggests the use of *Pinus* spp. resins in the funerary pigment mixture; although the pine resources are not locally available for many sites in the Maya lowlands, some researchers propose that pinewood may have been exchanged as trade goods during Maya prehistory [19].

**Figure 4:**
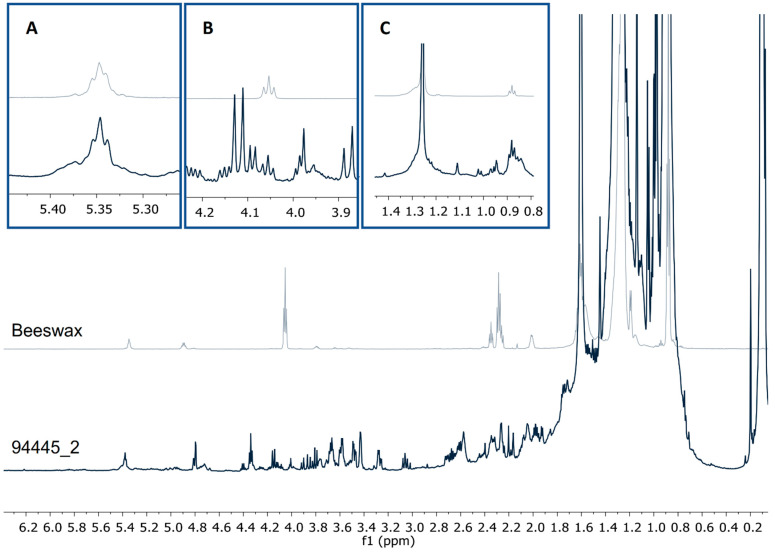
^1^H-NMR profiles (CDCl_3_, 600 MHz) from a pigment sample and beeswax showing the signals from (**A**) vinylic protons δ5.35, (**B**) ester 1° alcohol δ4.04, t, and (**C**) methyl protons δ0.88, t.

The presence of beeswax in the funerary pigment samples could be explained by the presence of four signals corresponding to a methyl group (δ0.88, t), methylenes (δ1.25), an esterified hydroxymethylene (δ4.04, t), and a vinylic proton (δ5.35) (Figure 4) [20,21]. To the best of our knowledge, this is the first time that beeswax is reported as a constituent of funerary pigments, although, its presence can be explained by its being commonly used in the preparation of ointments, agglutinants, and adhesives [21,22]. Furthermore, the use of plant resins and natural materials as beeswax in funerary rituals in funerary rituals has been well documented in both the ancient Egyptian mummification process [23], and in the mortuary rites of the Romans [24].

The results of this investigation reveal the complexity of the funerary pigments used by the Maya groups. The identification of additional metabolites from larger sample of funerary pigments, as well as the collection of resins present in the pigments to confirm the identity of components identified preliminarily, is currently underway.

### Limitations of the Study

Other than ^1^H-NMR analysis ^13^C-NMR and two-dimensional (2D-NMR) studies would help unravel and identify additional metabolites, as well to confirm the components found in the present study. However, given the lower amount of sample it was firstly necessary to develop a method for sample extraction and detection of the compounds, since ^13^C-NMR is much less sensitive than ^1^H-NMR and, owing the low ^13^C abundance in the samples, this study require several hours when compared to a common ^1^H-NMR recording (~4 min).

## 3. Materials and Methods

### 3.1. General Experimental Procedures

Solvent evaporation was carried under reduced pressure using a Yamato RE301 Rotary Evaporator (Tokyo, Japan). ^1^H-NMR experiments were carried out at 25 °C on a Varian-Agilent AR Premium Compact spectrometer (Santa Clara, CA, USA) at 599.77 MHz. Chemical shifts were referred to TMS (δ0.0). Dichloromethane (CH_2_Cl_2_) was purchased from Jalmek (San Nicolás de los Garza NL, México). Deuterated chloroform (CDCl_3_) and triterpene standards were purchased from Sigma-Aldrich (St. Louis, MO, USA).

### 3.2. Funerary Pigments Extraction

A total of 11 pigment samples belonging to seven different archaeological contexts of Xcambó were extracted and analyzed (Table 1). Two methods of sample extraction were evaluated because of limitations in the amount of material: in the first method, the pigment was scraped from the bone segment and the resulting powder was extracted (30 min) with 700 µL of CDCl_3_ using a sonicator (Branson, 3510). After 15 min, the supernatant was transferred to a 5 mm NMR tube. The second method involved the sonication of the bone segment in CH_2_Cl_2_, followed by concentration of the extract in a rotary evaporator and dissolving the residue in 700 µL of CDCl_3_, before transferring the samples to a 5 mm NMR tubes.

### 3.3. Resins and Triterpenoid Standards

The white copal (*Bursera* spp.) and storax (*Styrax* spp.) resins were acquired from a local market as, while the ocote resin was obtained by heating the corresponding wood segment (*Pinus* spp.) from Ocote Campirano^®^. The resin of chaká (*Bursera simaruba*) was collected directly from a tree at CINVESTAV—Unidad Merida, and the sample of beeswax was kindly donated by the Facultad de Medicina Veterinaria y Zootecnia, Universidad Autónoma de Yucatán. Part of these materials were kept in order to establish a local library of resins. A portion of each material (~20 mg) was dissolved in 700 µL of CDCl_3_. Separately, commercial samples of lupenone, lupeol, α-amirenone, α-amyrine, β-amyrin acetate, β-amyrenone, β-amyrine, batilol, botulin, and stigmasterol, were re-dissolved in 700 µL of CDCl_3_. All the samples were transferred to a 5 mm NMR tubes.

### 3.4. Generation of the ^1^H-NMR Profiles

The measurements were conducted with the s2pul sequence. The relaxation delay was 1.0 s, and the acquisition time was 3.0 s. The profiles of the funerary pigment samples were the result of 15,000 scans, while the resin and triterpenoid profiles were obtained by 64 scans with data collected into 64k data points. Each free induction decay (FID) was zero-filled to 128k data points. Prior to Fourier transformation, a Gaussian window function with a line broadening factor of 0.2 Hz was applied. The resulting spectra were manually phased, and baseline corrected using MNova 12.0 (Mestrelab Research S.L.).

## 4. Conclusions

In the present work the ^1^H-NMR was evaluated as an exploratory technique that allowed the detection and preliminary characterization of organic components in the funerary pigments from Xcambó. In addition, two methods of pigment extraction were compared, thus identifying the sonication of the bone segment as the one with the best yield of extraction and less esthetical damage. This study opens new perspectives for the study of bone pieces without affecting their structure.

The study of the aggregates in the funerary pigments gives an insight about the consistency of the ancient product and its consequent mode of application. The results indicated that the pigment was combined with resins which must have been melted for mixing, however, such resin hardens quickly when removed from the heat, making it difficult to manipulate (Berdan, 2007). Therefore, the finding of wax in samples from Xcambó reflects the use of a necessary material in the recipe, in addition to a technique of preparation and application: The wax was the excipient and emollient, the base of the pigment; while the resin bound, its oils provided aroma and, in certain cases, also an antibacterial quality. Exposure to heat allowed the mixing of the ingredients, which could be obtained separately or in a single product. This is relevant if we consider that some resins and the pigment itself were not obtained in Xcambó. Further characterization by 2D-NMR analysis would expand the information about additional components, allowing to suggest specific resin-producer species and the possible source of these.

## Figures and Tables

**Table 1:** Archaeological pigment samples from Xcambó used in the study.

**Burial**	**Sample**	**Pigment Recovery Method**
**Patio IX 349**	94409_1	scraping
	94409_1	scraping
**NE-7A/76**	94289_1	scraping
	94289_2	scraping
**Patio IX/337**	94402_1	scraping
	94402_2	scraping
**NO-4/96**	94544_1	scraping
**NE-7A/34**	94046_1	scraping
**NE-21A/6**	94228_1	scraping
**NE-7A/80**	94445_1	sonication of the bone segment
	94445_2	sonication of the bone segment

## Data Availability

The data presented in this study are available on request from the corresponding author.

## Supplementary Materials

Figure S1: Location of the Xcambó archaeological site in the Yucatan Peninsula (Taken from Maggiano et al., 2008); Figure S2: Photography showing the presence of red pigments in the funerary contexts of (A) The Red Queen of Palenque, (B) Burial NO-4/96 from Xcambó; Figure S3: ^1^H-NMR (CDCl_3_, 600 MHz) profiles of the pigments corresponding to diverse archaeological contexts of Xcambó and used in the present study.

## Sample Availability

Samples of the compounds are not available from the authors.
