# Supplementary material for: An Insight to the Composition of Pre-Hispanic Mayan Funerary Pigments by 1H-NMR Analysis
Source: Molecules. 2021 May 17;26(10):2972. doi: 10.3390/molecules26102972 (PMC8157014; doi:10.3390/molecules26102972)
Supplement: Supplementary file 1 [file molecules-26-02972-s001.zip › molecules-1195588-supplementary.pdf]

Article

An Insight to the Composition of Pre-Hispanic Mayan Funerary Pigments by ^{1}H-NMR Analysis

Kadwin J. Pérez-López [1], Vera Tiesler [1], Patricia Quintana [2], Emanuel Hernández-Nuñez [3,*] and Gloria I. Hernández-Bolio [2,*]

[1] Facultad de Ciencias Antropológicas, Universidad Autónoma de Yucatán, 97305 Mérida, Yucatán, Mexico; kadwinp@gmail.com (K.J.P.-L.); vtiesler@correo.uady.mx (V.T.)
[2] Centro de Investigación y de Estudios Avanzados, Departamento de Física Aplicada del Instituto Politécnico Nacional, Unidad Mérida, 97310 Mérida, Yucatán, Mexico; pquint@cinvestav.mx
[3] CONACYT, Centro de Investigación y de Estudios Avanzados del Instituto Politécnico Nacional, Departamento de Recursos del Mar, Unidad Mérida, 97310 Mérida, Yucatán, Mexico
* Correspondence: emanuel.hernandez@cinvestav.mx (E.H.-N.); gloria.hernandez@cinvestav.mx (G.I.H.-B.); Tel.: +52-(999)-105-5211 (E.H.-N.); +52-(999)-942-9400 (G.I.H.-B.)

Abstract: The funerary rites of particular members of the pre-Hispanic Mayan society included the pigmentation of the corpse with a red color. In order to understand this ritual, it is first necessary to identify the constituents of the pigment mixture and then, based on its properties, analyze the possible form and moment of application. In the present approach, ^{1}H-NMR analysis was carried to detect organic components in the funerary pigments of Xcambó, a small Maya settlement in the Yucatan Peninsula. The comparison of the spectra belonging to the pigment found in the bone remains of seven individuals, and those from natural materials, led to the identification of beeswax and an abietane resin as constituents of the pigment, thus conferring it agglutinant and aromatic properties, respectively. The ^{1}H-NMR analysis also allowed to rule out the presence of copal, a resin found in the pigment cover from paramount chiefs from the Mayan society. Additionally, a protocol for the extraction of the organic fraction from the bone segment without visible signs of analysis was developed, thus broadening the techniques available to investigate these valuable samples.

Keywords: abietane diterpenoids, beeswax, bone remains, resins

Citation: Pérez-López, K.J.; Tiesler, V.; Quintana, P.; Hernández-Nuñez, E.; Hernández-Bolio, G.I. An Insight to the Composition of Pre-Hispanic Mayan Funerary Pigments by ^{1}H-NMR Analysis. *Molecules* **2021**, *26*, 2972. https://doi.org/10.3390/molecules26102972

Academic Editors: Bruno Botta, Deborah Quaglio, Cinzia Ingallina and Andrea Calcaterra

Received: 8 April 2021
Accepted: 12 May 2021
Published: 17 May 2021

Publisher's Note: MDPI stays neutral with regard to jurisdictional claims in published maps and institutional affiliations.

Copyright: © 2021 by the authors. Licensee MDPI, Basel, Switzerland. This article is an open access article distributed under the terms and conditions of the Creative Commons Attribution (CC BY) license (https://creativecommons.org/licenses/by/4.0/).

SUPPLEMENTARY MATERIALS

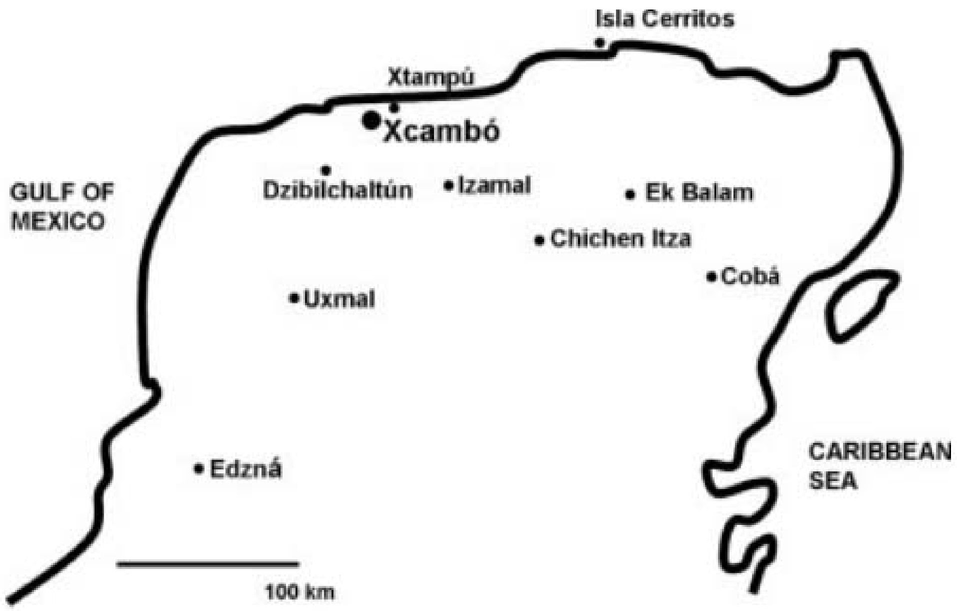

Figure S1. Location of the Xcambó archaeological site in the Yucatan Peninsula (Taken from Maggiano et al., 2008).

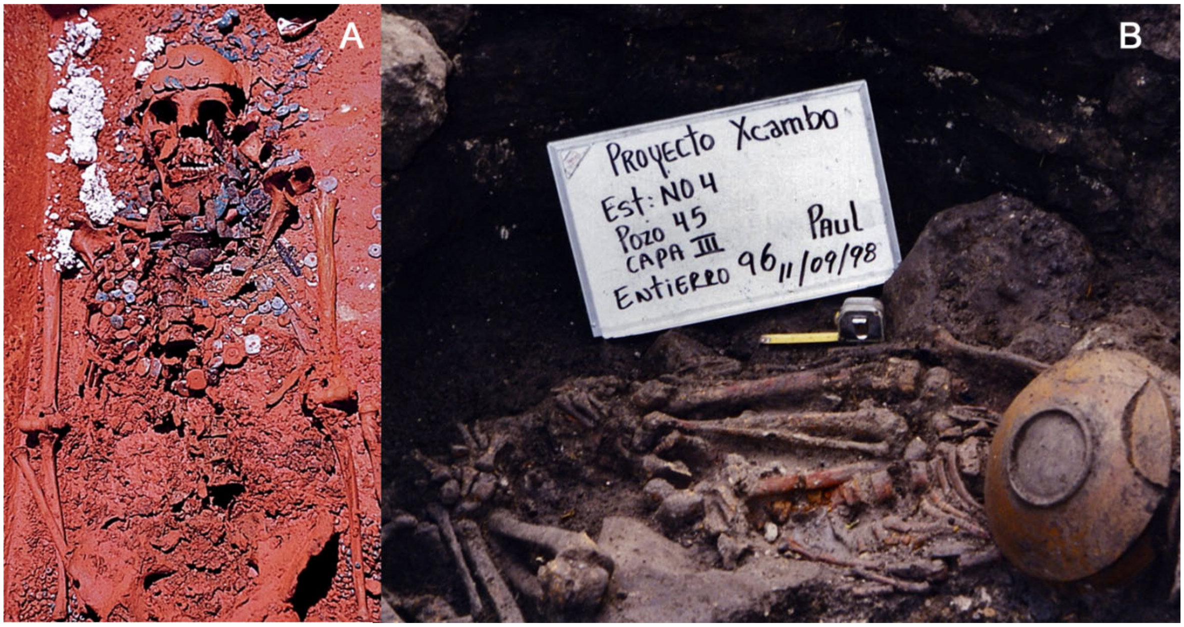

Figure S2. Photography showing the presence of red pigments in the funerary contexts of A) The Red Queen of Palenque, B) Burial NO-4/96 from Xcambó. (Photo credits: Javier Hinojosa, Proyecto Arqueológico Xcambó).

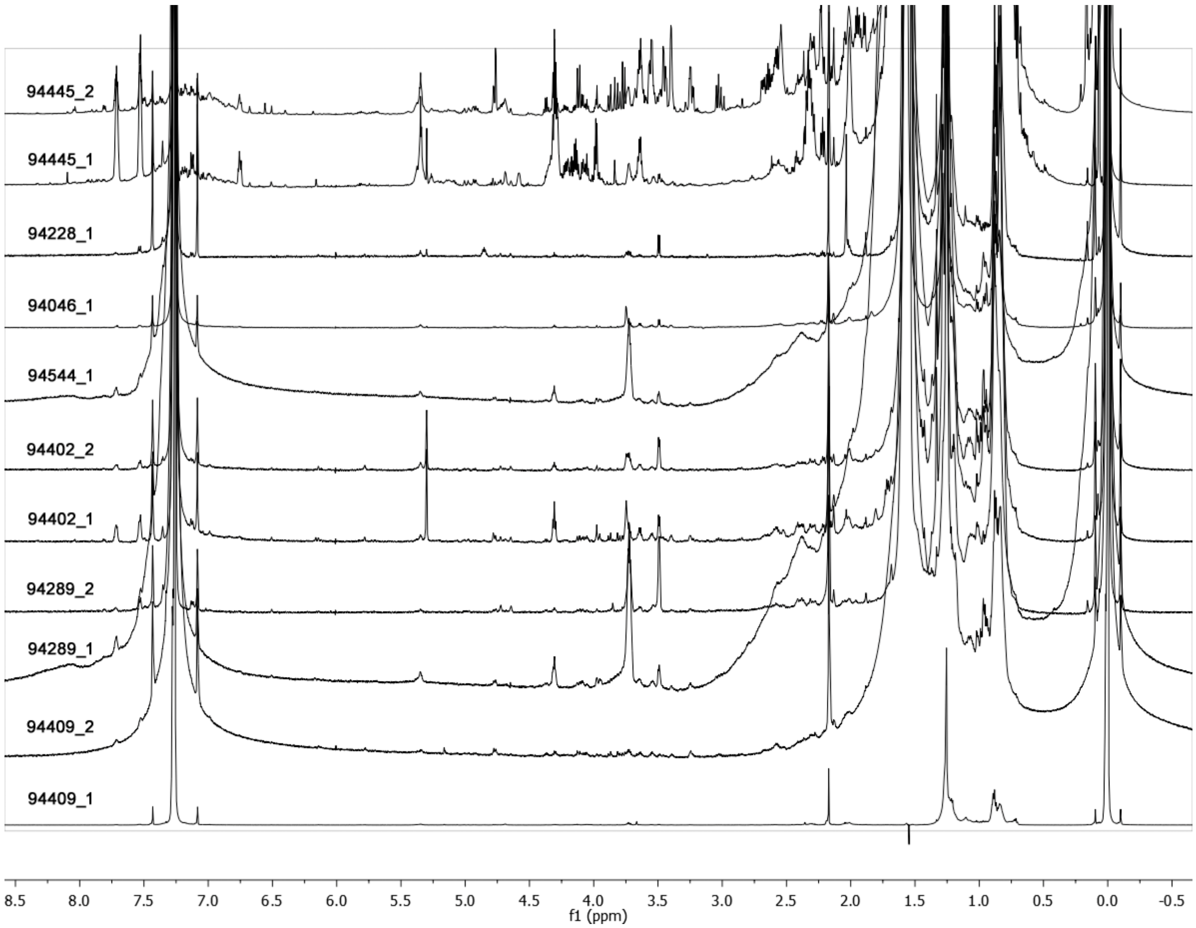

Figure S3. ^{1}H-NMR ($CDCl_3$, 600 MHz) profiles of the pigments corresponding to diverse archaeological contexts of Xcambó and used in the present study.
